# Pupil Size as a Gateway Into Conscious Interpretation of Brightness

**DOI:** 10.3389/fneur.2018.01070

**Published:** 2018-12-13

**Authors:** Irene Sperandio, Nikki Bond, Paola Binda

**Affiliations:** ^1^The School of Psychology, University of East Anglia, Norwich, United Kingdom; ^2^Department of Translational Research and New Technologies in Medicine and Surgery, University of Pisa, Pisa, Italy; ^3^Institute of Neuroscience, Consiglio Nazionale delle Ricerche (CNR), Pisa, Italy

**Keywords:** pupillometry, pupillary constriction, high-level visual processing, visual awareness, brightness

## Abstract

Although retinal illumination is the main determinant of pupil size, evidence indicates that extra-retinal factors, including attention and contextual information, also modulate the pupillary response. For example, stimuli that evoke the idea of brightness (e.g., pictures of the sun) induce pupillary constriction compared to control stimuli of matched luminance. Is conscious appraisal of these stimuli necessary for the pupillary constriction to occur? Participants' pupil diameter was recorded while sun pictures and their phase-scrambled versions were shown to the left eye. A stream of Mondrian patterns was displayed to the right eye to produce continuous flash suppression, which rendered the left-eye stimuli invisible on some trials. Results revealed that when participants were aware of the sun pictures their pupils constricted relative to the control stimuli. This was not the case when the pictures were successfully suppressed from awareness, demonstrating that pupil size is highly sensitive to the contents of consciousness.

## Introduction

Increments or decrements of light are associated with pupillary constrictions or dilations, respectively. This is known as the pupillary light reflex, and has been traditionally considered as a low-level mechanism that simply regulates the amount of light that enters the eye to optimize vision. However, since pupillometry—i.e., the measurement of the diameter and rate of reactivity of the pupil—was introduced more than 50 years ago, it soon became evident that pupillary responses can be used to index cognitive operations, such as thinking and emotional processing [(1, 2) for a review see (3)]. More recently, it has been argued that high-level visual processing, including attention, mental imagery, and contextual modulation, can also influence pupillary responses under conditions of constant retinal illumination, demonstrating that the pupil diameter is not solely determined by physiological factors [for reviews, see (4, 5)]. For example, it has been shown that covert shifts of attention to brighter surfaces cause pupillary constrictions (6–8) and that similar changes in pupil diameter can be induced even in the absence of visual stimulation by asking participants to mentally visualize a bright scene (9). By the same token, the pupil constricts in response to visual illusions of brightness (10) and stimuli that evoke the idea of bright objects, like pictures of the sun (11, 12) or words conveying brightness (13). Another line of experiments showing pupil changes in conditions of constant retinal stimulation examined the phenomenon of binocular rivalry between stimuli of different luminance. The typical finding is that pupil size follows the dominant percept, with a relative constriction when the brighter stimulus dominates conscious perception (14–16), and an attenuation of pupillary responses to light flashes when these were presented to the suppressed eye (14, 17–19). There is also a line of experiments demonstrating the importance of cortical signals, and specifically signals from the occipital visual cortex, for modulating the pupillary response to light. For example, pupil perimetry (measurement of the pupillary response to light stimuli located at different loci across the visual field) has provided clear evidence of reduced or absent pupillary light reflex in the blind visual area of patients with lesions to the occipital lobe [e.g., (20–23)].

Taken together, these findings provide compelling evidence in support of the view that the pupillary light reflex is sensitive to top-down modulation. This suggests that pupil light responses may be used as a read-out of the idiosyncrasies of visual perception—a simple, non-invasive, objective, and quantitative measure of our attentional biases, our illusion susceptibility, our ability to use contextual information etc., and an initial success of this strategy has recently been reported (24). However, before these exciting avenues can be explored, it is necessary to demonstrate that top-down effects on pupil response do in fact reflect the contents of visual awareness. One possibility to address this issue is to look for correlations between perceptual (e.g., brightness) judgments and pupillary responses (9, 25). Here we took a more radical approach and tested whether one such top-down effect requires visual awareness of the stimuli—and whether it is absent when stimuli are not consciously perceived.

The perceptual visibility of the stimuli, specifically pictures of the sun (11), was manipulated by means of continuous flash suppression [CFS; (26)], a widely used technique that enables to reliably erase stimuli from visual awareness for extended periods of time [for reviews, see (27, 28)]. During CFS, a static image presented to one eye is rendered invisible by ever-changing Mondrian patterns displayed to the other eye. This interocular suppression technique seems to be particularly effective at disrupting high-level visual processing completely [for a review, see (28)]. A particularly clear case can be made from adaptation studies. Aftereffects specific to complex motion [e.g., (29)], facial expression of emotions [(30); but see (31)], and subordinate information about faces, such as gender or race (32), all of which require higher order visual processing, were abolished when adapters were suppressed from awareness by CFS. In contrast, aftereffects specific to low-level stimulus attributes, such as orientation [e.g., (33)] and contrast [e.g., (34)], were only attenuated by CFS. Evidence from other paradigms, such as priming and braking-CFS, is more mixed. A suppressed stimulus is more likely to break CFS and come back to awareness when it is familiar and provided with emotional values [e.g., (35, 36)], suggesting that at least some form of high-level information may be processed even when the stimulus is suppressed from awareness [for a review, see (37)]. Similarly, a subliminal form of priming by stimuli made invisible by CFS, may in some cases be observed for numerosity [e.g., (38)], object category [e.g., (39)] and emotional content [e.g., (40)]; however, in other cases, priming effects for complex stimulus features, such as word stimuli (41), emotional faces (42), and threatening animal stimuli (43), vanish completely when the prime is rendered invisible by CFS. Thus, the literature on the effects of CFS on low- and high-level visual processing is mixed, suggesting that processing of many stimulus features can take place outside of conscious awareness. Nevertheless, high-level visual properties are likely to succumb suppression [e.g., (28, 44)], at least more than low-level simple visual features, which continue to adapt and shape perceptual processes quite irrespectively of awareness.

With the support of this literature, here we aimed to study how CFS would affect the pupillary response to sun image (vs. their phase scrambled versions). If—as argued before—the relative pupillary constriction evoked by the sun images depends on high-level visual processing, we predict that it should be abolished, or at least diminished, under CFS.

## Methods

### Participants

Twenty-four participants (16 females, all right handed) ranging in age from 18 to 27 years (*M* = 20.48, *SD* = 2.24) with normal or corrected-to-normal vision, took part in the experiment. This sample size was deemed to be appropriate to attain a moderate effect size with α = 0.05 and power = 0.80, according to calculations performed in G^*^Power (45). Two participants were excluded due to technical issues and an additional participant was excluded due to poor stereoacuity, as assessed using the Frisby stereotest (Clement Clarke International Ltd, Essex, UK). This resulted in a final sample size of 21 participants (13 females) with an age range of 18–27 years (*M* = 20.42, *SD* = 2.03). Participants received either 2 study credits or £5 for their time. Written consent was obtained prior to testing. All procedures were approved by the School of Psychology Research Ethics Committee of the University of East Anglia and were carried out in accordance with the Declaration of Helsinki.

### Apparatus and Stimuli

The experiment was programmed in E-Prime 2.0 (Psychology Software Tools, Pittsburg, PA, USA) on a Viglen DQ77MK, running Windows 7. Stimuli were presented on a 16-inch Dell monitor with a resolution of 1,280 × 1,024 pixels and a refresh rate of 60 Hz. Participants were seated in front of the computer monitor in a dark room with their head fixed on a chin rest at a distance of 57 cm and viewed the stimuli through a mirror stereoscope. A divider (i.e., a sheet of cardboard) was placed between the stereoscope's midline and the center of the monitor to ensure that images displayed on each half of the monitor would be seen by each eye separately (46). Participants wore SMI™ (SensoMotoric Instruments) eye tracking glasses to measure their pupil diameter (see “Eye Tracking” for details).

Stimuli consisted of 13 different pictures of the sun and their phase-scrambled counterparts of matched luminance, as developed by Binda et al. (11) (available at: http://faculty.washington.edu/somurray/PupilSun/). The Supplementary material reports an analysis of the luminance profile of all images as a function of distance from image center (i.e., eccentricity, since fixation was maintained at image center). Across all images, there was a tendency for the sun images to have higher luminance than their phase scrambled versions near the center (although luminance was always lower than that of the pre-stimulus white screen), but this was not always the case (it is possible to select a subsample of pictures with matched luminance profiles, where additional analyses of the pupillary responses can be performed, see Figures S2, S3).

**Figure 1:**
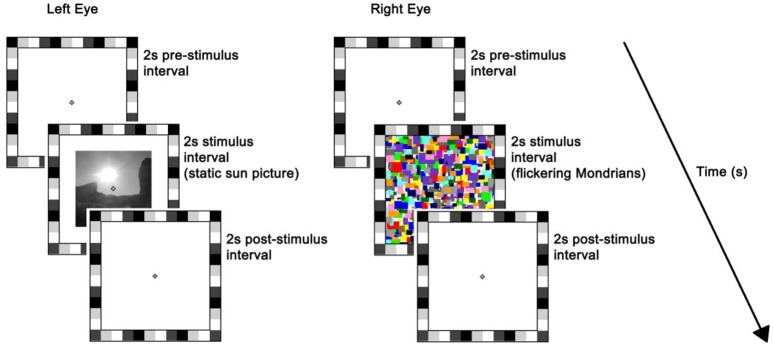
Trial sequence and timing. At the beginning of each trial, a white background of maximum luminance was presented for 2 s (i.e., pre-stimulus interval). This was followed by a 2 s stimulus interval where a static picture (sun or scrambled version) was displayed to the left-eye while flickering Mondrians (CFS; as depicted here) or a blank background (No_CFS) were displayed to the right-eye. Finally, a blank screen was presented for 2 s during the post-stimulus interval. The flickering Mondrians successfully suppressed the static image in ~50% of trials on average, whereas images were always seen when accompanied by the blank.

The stimulus was presented on the left half of the monitor. The rival stimulus consisted of a series of high-contrast Mondrian patterns with a flicker rate of 10 Hz, which were displayed to the right half of the monitor (26). The series of Mondrian patterns consisted of five distinct images cycling in a sequential order with individual frames refreshing every 100 ms (mean luminance = 70.84 cd/m^2^). Both static and flickering stimuli were preceded and followed by a plain white background of maximum luminance, i.e., 196.30 cd/m^2^, and subtended 7° × 7° of visual angle. A 1°-thick frame of various grayscale squares was placed around the stimuli to assist binocular alignment and a white small fixation cross was centered in each eye's stimulus to aid stable fixation (Figure 1). Participants' response was recorded by means of a keyboard.

### Procedure

Prior to the beginning of the experiment, two frames with a fixation cross in each were presented dichoptically to the eyes and participants were instructed to adjust the stereoscope's mirrors until the left and right stimuli were correctly aligned and fused. In addition, to ensure that binocular fusion was maintained throughout the experiment, the same calibration stimuli were presented at the beginning of each trial and participants were asked to initiate the trial by pressing the spacebar on the keyboard only after they perceived a single frame and fixation cross.

To manipulate the perceptual visibility of the stimuli, participants were tested both in CFS and no_CFS conditions. In the CFS condition, the right eye was presented with a continuous stream of Mondrian images while static pictures of the sun or their scrambled versions were displayed to the left eye. Under this condition, the CFS mask typically renders perceptually invisible the static images for prolonged periods of time (26). In the no_CFS condition, the Mondrians were replaced by a blank background. The removal of the CFS mask should make the left-eye stimulus easily visible to the participants [e.g., (47)].

Each trial lasted for 6 s, which consisted of: (a) 2-s blank *pre-stimulus interval* where participants' eyes were exposed to the maximum luminance of the monitor; (b) 2-s *stimulus interval* where a static picture (sun or scrambled version) was displayed to the left-eye while flickering Mondrians (CFS) or a blank background (no_CFS) were displayed to the right-eye; (c) 2-s *post-stimulus interval* where the monitor returned to the maximum luminance (11). Participants were discouraged from blinking or making saccades over the entire duration of the trial but they were allowed to do so in between trials.

To ensure that stimuli were truly suppressed from conscious perception under the CFS condition, participants were asked at the end of each trial to report whether or not they saw an image besides the CFS mask (i.e., failure of suppression) by pressing designated keys on the keyboard. These unsuccessful trials were labeled as “failed CFS.” In order to compare pupil traces in failed and successful CFS trials (characterized by identical stimulation but different conscious percept), we aimed to collect a significant amount of “failed CFS” trials, ideally as many as the successful ones. To this end, we chose to flash the sun/scrambled image abruptly, rather than gradually ramping it in, since flashing the stimulus is known to encourage CFS-breaking [e.g., (48)].

The experiment consisted of 52 trials in total, namely 2 suppression conditions × 2 image types × 13 repetitions. The suppression conditions (i.e., CFS vs. no_CFS) were tested in two separate blocks. The order of these two blocks was counterbalanced across participants. Within each block, trials were presented in a random fashion.

### Eye Tracking

SMI™ eye tracking glasses registered pupil diameter binocularly at a sampling rate of 60 Hz. A 3-point calibration was performed at the beginning of each block. Time points with impossible pupil size (i.e., exceeding the range 2–8 mm) were considered as signal losses and removed from the analysis. To measure the change in pupil diameter evoked by the static pictures, individual data were baseline-corrected against a 500-ms window preceding the stimulus presentation. The time course of the pupillary response was determined by averaging baseline-corrected data in 250-ms bins (25 data points). To allow comparisons across conditions, an average of the baseline-corrected data during the last second of stimulus presentation was also calculated (this window was selected based on previous data, as the interval where the difference across image types is expected to be the largest, see 11).

### Data Analysis

A two-way repeated measures analysis of variance (ANOVA) was carried out on average baseline-corrected pupil diameter during the last second of stimulus presentation, to evaluate the main effects of Condition (no CFS vs. CFS) and Image Type (sun vs. phase-scrambled), and their interaction. To establish if changes in pupil diameter could predict individual differences in visual awareness of the pictures, we further analyzed the effect of Image Type separately in CFS trials where pictures were successfully suppressed or where they could still be seen by the participant. For this analysis, we relied on a Linear Mixed Model approach, motivated by the considerable sample size variability across subjects (due to the variability of the CFS success). In this approach, individual trials from all subjects are compared with a model comprising both the effect of experimental variables (“fixed effects”) and the variability across participants (“random effects”). Fixed effects were coded as categorical variables Image type (sun vs. phase-scrambled) and Visibility Condition (no-CFS, failed-CFS and successful CFS). Random effects were coded by allowing subject-by-subject variations of both the slope and intercept for each of the fixed effects. An additional analysis reported in the Supplementary material combines the two approaches described above and directly compares pupil responses when the sun or scrambled images were seen (no-CFS and failed-CFS trials) or unseen (successful CFS trials), either considering all images or a subset of sun/scrambled images with matching luminance profiles.

For all analyses, we used standard MATLAB functions provided with the Statistics and Machine Learning Toolbox (R2015b, The MathWorks). Specifically, the function “fitlme (data, model)” fit the linear-mixed model to the data, yielding an object “lme” with associated method “anova” that returns *F* statistics and *P*-values for each of the fixed effect terms. The function “fitrm (data, model)” fit the ANOVA for repeated measures, returning *F* statistics, degrees of freedom and associated *P*-values. Standard *t*-test functions were complemented with Bayes Factors estimations, using the “Bayes Factors” toolbox for Matlab available online at https://figshare.com/articles/Bayes_Factors_Matlab_functions/1357917. All reported *p*-values were based on two-tailed criteria.

## Results

We tracked changes in pupil diameter induced by the presentation of pictures of the sun and their scrambled version (11) and manipulated the perceptual visibility of the stimuli by means of CFS. The CFS mask successfully suppressed the unchanging image in 58.29% (s.e.m. 7.17%) of trials, while images were constantly visible under the no_CFS condition. The average baseline pupil size (during blanks, when screen luminance was maximum) was 3.66 mm (s.e.m = 0.11 mm); pupil baseline values were tightly distributed around this value, and never exceeded the 2.5–6.5 mm range, ensuring that our measurements were clear of the physiological limits of pupil diameter, where mechanical factors could artefactually reduce pupil size variability.

**Figure 2:**
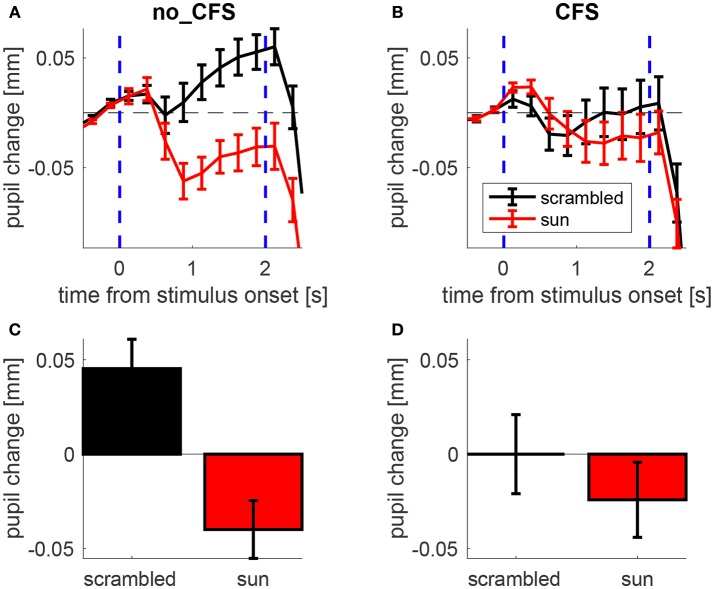
Pupillary response to sun pictures and their phase-scrambled versions, under no_CFS (visible), and CFS conditions. **(A,B)** Baseline-corrected pupil diameter (i.e., pupil change) as a function of time from stimulus onset. **(C,D)** Mean pupil change during the last second of the stimulus interval. Error bars represent standard error of the mean (s.e.m) across our 21 observers.

Figure 2A illustrates the time course of the pupillary response averaged across participants while mean pupil changes during the stimulus presentation is shown in Figure 2B.

A 2 × 2 ANOVA for repeated measures was carried out on the mean pupil size during the stimulus interval (shown in Figures 2C,D) with Condition (no-CFS vs. CFS) and Image Type (sun vs. scrambled) as main factors, revealing a significant interaction (*F*_1,20_ = 12.835, *p* < 0.01). The statistical significance of the interaction term means that the pupil difference between sun and scrambled images varies across conditions. This indicates that the CFS procedure was able to modulate the “sun-pupil effect” and suggests that the level of conscious awareness of the images is important for determining the pupil response they evoke. Note that the same conclusions hold when analyzing pupillary responses in trials when the sun/scrambled images were seen or unseen (Figure S1, collapsing no_CFS trials and trials in which CFS failed to suppress awareness of the images, and comparing them with trials in which CFS was successful in suppressing awareness). The conclusions also hold when analyzing only a subset of trials where both the average luminance and the spatial profile of luminance are matched between sun and scrambled images (Figures S2, S3).

**Figure 3:**
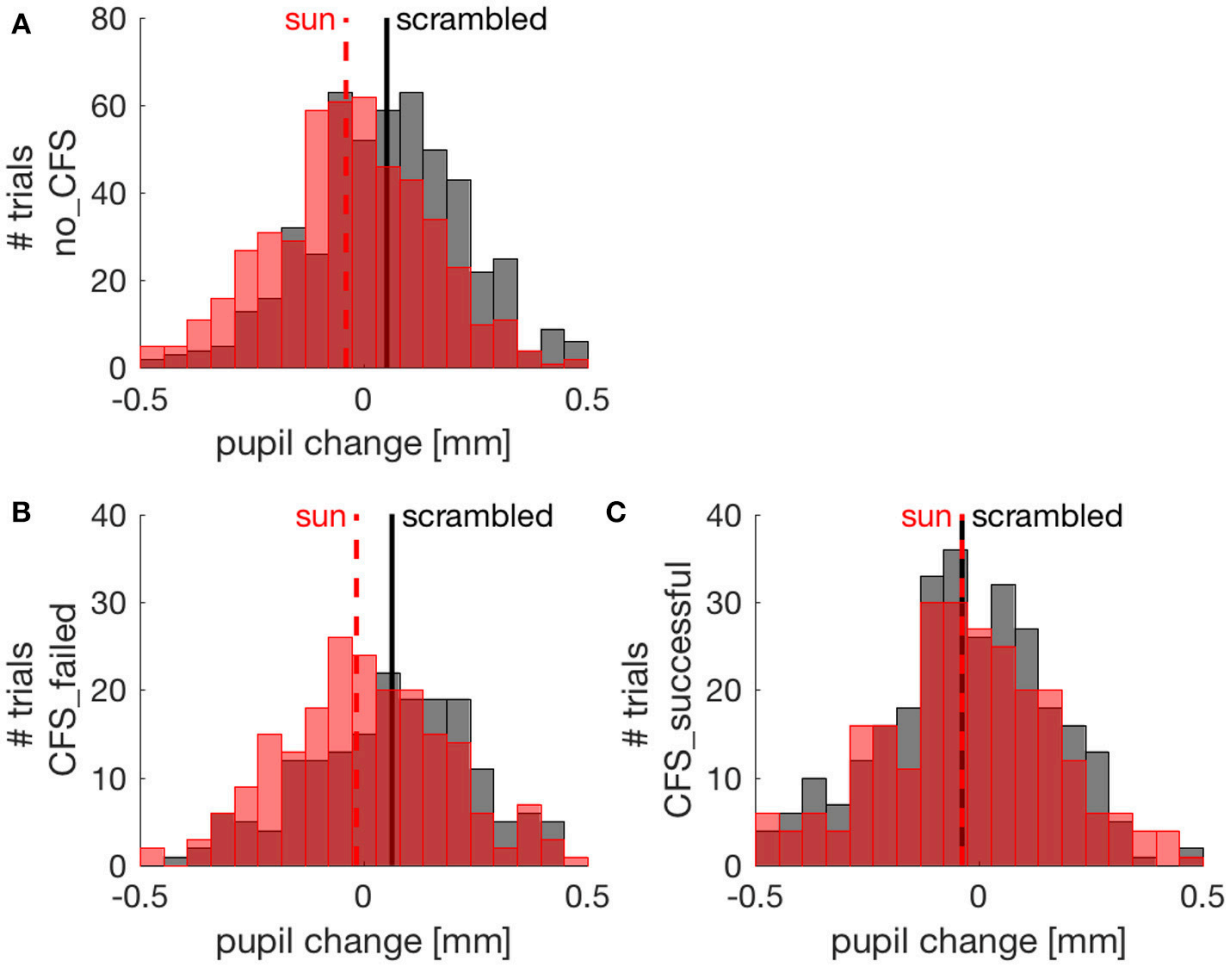
Pupil changes (relative to the pre-stimulus baseline) in individual trials (pooled across participants). Red and black distributions show pupil responses to sun and phase scrambled pictures, respectively, with their means indicated by vertical lines. **(A)** trials from the no CFS condition; **(B)** trials where CFS failed to suppress awareness of the picture; **(C)** trials where CFS successfully made the sun/scrambled pictures unseen.

To further investigate the effect of suppressing images from conscious awareness, we focused on the CFS condition and analyzed pupil responses separately in trials where CFS failed to suppress awareness of the sun/phase-scrambled pictures and where it succeeded in suppressing pictures visibility. Because different participants contributed an uneven number of trials, this analysis was conducted with a Linear Mixed Model approach (see methods). Figure 3 shows the distribution of pupil responses when the sun pictures or the phase-scrambled images were displayed for the no_CFS condition (panel A), and separately for trials where CFS was successful at making the pictures invisible (panel C) and trials where pictures remained visible despite CFS (panel B). In the latter case, like in the no_CFS condition, there was a clear and reliable difference between pupil responses to the sun pictures and their phase-scrambled versions. In line with this, the Linear Mixed Model analysis revealed a significant interaction (*F*_(2, 1970)_ = 7.786, *p* < 0.001) between the factors “Image Type” (sun vs. phase-scrambled) and “Suppression Condition” (no_CFS vs. failed CFS vs. successful CFS). The same significant interaction holds when selecting only CFS trials, failed and successful (*F*_(1, 956)_ = 4.842, *p* < 0.05) indicating that the sun-scrambled pupil difference depends on the awareness of the images. *Post-hoc t*-tests indicated a significant effect of Image type in the no-mask [two-sample *t*-test: *t*_(510, 506)_ = 6.71, *p* < 10^−5^] and failed CFS [*t*_(186, 214)_ = 3.50, *p* < 0.001] conditions. However, there was no reliable difference between pupil responses to the sun and phase-scrambled pictures in trials where they were not consciously perceived, due to successful CFS [*t*_(295, 265)_ = 0.01, *p* = 0.994]. For each of these *t*-tests, we computed the JZS Bayes Factor (49), which quantifies the amount of evidence against or in favor of the null hypothesis (i.e., that sun and phase-scrambled pictures evoke equal pupil responses): a BF smaller than 0.3 is strong evidence in favor of the null hypothesis; a BF larger than 3 is strong evidence against it. In the no-mask and the failed CFS condition, Bayes Factors were >30. In the successful CFS condition, however, the Bayes Factor was 0.094: strong evidence in support of the null hypothesis, or equal pupillary response to the sun and scrambled images.

Complementary to these *post-hoc* tests is another set of comparisons assessing the effect of CFS on pupillary responses to each image type (sun and scrambled). These indicate that the pupil dilation evoked by scrambled images was significantly reduced in successful CFS trials compared to failed CFS trials (*t*_(186, 295)_ = 4.71, *p* < 10^−5^, BF = 3903), whereas the pupil response to sun images was the same (*t*_(204, 265)_ = 1.00, *p* = 0.317, BF = 0.166). Due to a limitation of the experimental design, this result does not lend itself to an unequivocal interpretation (as discussed below).

## Discussion

A growing body of evidence shows the role that extra-retinal factors exerts on the pupil diameter, challenging the notion that the pupillary light response is merely a reflex. The aim of the current study was to determine whether these modulations require visual awareness. In agreement with previous research (11, 12), we observed pupil constrictions to pictures of the sun relative to their phase-scrambled versions. However, this effect was only present when participants were aware of such stimuli, namely when the mask was replaced by a blank background (no_CFS) or when the stimuli broke through suppression and became consciously visible (failed_CFS). The effect disappeared when stimuli were made invisible (successful_CFS). Importantly, any potential difference in the luminance profile of the stimuli cannot account for the effect, implying that retinal and subcortical processing alone are insufficient to explain changes in pupil response. Instead, the pupil needs conscious (high-level) processing to be able to distinguish between sun and phase-scrambled pictures. This finding is in line with the large literature on CFS, showing that suppressing a stimulus from conscious awareness limits its perceptual processing, especially for complex high-level stimulus properties (28, 37).

The hypothesis of a high-level modulation of pupil diameter is supported by numerous studies demonstrating changes in pupil response during high-level cognition, including spatial attention (6–8, 50), imagery (9), memory (51), decision-making (52), contextual (10–12), and semantic processing (13). Relevant to the present work is the observation of pupil modulations during binocular rivalry of stimuli with different luminance, whereby pupillary dilations were associated with perceptual transitions from bright to dark stimuli, and pupillary constrictions with transitions from dark to bright stimuli (15). Similar to Naber et al. (15), we found that under constant retinal illumination, pupil size adjusts according to the dominant percept. However, in our case, pupil size is independent of actual luminance of the dominant image [as was in Naber et al. (15)] but depends on high-level visual analyses producing a differential pupil response to pictorial representations of a high-luminance object (the sun) vs. a meaningless image matched in luminance and contrast (scrambled).

Note that, when images were successfully suppressed from visual awareness, the pupillary response was dominated by constriction—not dilation, as could be expected if the constriction in response to the sun image was selectively suppressed. This finding lends itself to two explanations. The first, which is hard to interpret, is that CFS only affects the pupil dilation in response to the scrambled images, leaving the response to sun images unaffected. The second, which we deem more sound, is that successful CFS trials are associated with enhanced pupil constriction because the high-contrast Mondrian mask-pattern dominates perception in these trials. This is a very reasonable scenario, given that high contrast images are known to generate pupillary constriction, provided that they are cortically processed [as reviewed in Barbur (53)], and given previous evidence shows that, when different stimuli are presented to the two eyes, pupillary responses are primarily driven by the consciously perceived stimulus (15). This constriction response to the mask-pattern confounds the interpretation of the individual pupil traces in response to the sun and scrambled images, leading to our inability to establish whether CFS interferes more with the response to one or the other image type. However, this does not confound our ability to compare the sun-scrambled difference in pupil response across conditions, and affirms that this is reduced in successful CFS trials, implying that CFS hampers the signals that differentiate sun and scrambled images for the purpose of generating a pupillary response.

What are these signals, and how do they affect pupil control? The pupillary light reflex relies on a simple subcortical system: from the retina, luminance signals are relayed to the olivary pretectal nucleus, which activates the parasympathetic neurons of the Edinger-Westphal nucleus to induce pupillary constriction (54). Our findings along with several pupillometry studies lead to the suggestion that the pupilloconstrictor activity must incorporate input from a separate pathway: a brightness signal from the visual cortex, which is sensitive to the top-down effects described above [see also (55)]. This idea is further corroborated by a recent study on patients with Parinaud's syndrome (56), a rare condition following selective lesions of the subcortical pretectal area; although the pupillary reflex was depleted in these patients, remarkably their pupil size was modulated by attention. This indicates that pupil response may be regulated by multiple pathways, some of which are cortically-mediated. Together with the present results, this implies that pupil control incorporates information from relatively complex cortical visual processing. This conclusion is line with direct evidence from cortical lesion patients, who have atypical pupillary responses to light (e.g., 20–23) and to contrast, which are tightly linked to their residual (sometimes unconscious, e.g., blindisight) visual abilities (57).

Although a high-level cortical site appears to be the most likely origin for the signals controlling the pupil sun-scrambled differential response, we cannot exclude the possibility that both the perceptual suppression and the suppression of the pupil response in fact originate at an earlier site. Our two image categories (sun and scrambled) were matched in luminance and (for many images, see Figures S2, S3) in the gross spatial profile of luminance. However, many simple visual features were eliminated by the phase scrambling procedure, including local contrast at lines and edges (58). Further insight into the neural underpinning of this effect could be gained by creating alternative control images, through novel scrambling methods [e.g., (59)].

A note on the size of pupil modulations is in order. The pupil modulations we report here are 0.1 mm and less. These are similar in size to the effects of other perceptual and cognitive variables found to affect pupil size: while light responses are often in the range of 1 mm and more (2), 0.05–0.1mm is the typical size of pupil responses to equiluminant contrast (53), motion direction changes (60), spatial attention (6), and feature-based attention (61), implying that pupil modulations in this range can be reliably measured (with eye-tracking apparatus comparable to the one used here). Albeit measurable, 0.05–0.1 mm pupil change is very small compared to the full range of pupil size (2–9 mm). Appreciation of this point is important to guide speculations on the functional relevance of this and other cognitive and perceptual influences on pupil size. Some have argued that these influences could “optimize” the optics of the eye for specific perceptual and cognitive tasks, given that pupil diameter is known to affect the light adaptation state of the retina (62) and visual spatial resolution (63). However, there is no evidence that changing pupil diameter by a fraction of mm has any measurable consequence on vision. Thus, it is possible that the importance of these small pupil modulations does not lay in their impact on perception, but in their usefulness as indices to track the contents of perception or cognition. Specifically, here we have shown that pupil size is a sensitive and accessible index of visual awareness, which can precisely track the contents of consciousness on a trial-by-trial basis. As such, pupillometry may prove to be an important tool for the study of consciousness that could overcome methodological limitations of introspective reports when assessing perceptual experience.

## Author Contributions

IS and PB conceived the study and developed the study design. IS and NB built the experimental setup and developed the software for eye-tracking and stimulus presentation. NB performed data collection. PB analyzed the data. IS wrote the manuscript and PB provided critical revisions. All authors approved the final version of the manuscript for submission.

### Conflict of Interest Statement

The authors declare that the research was conducted in the absence of any commercial or financial relationships that could be construed as a potential conflict of interest. The handling editor declared a past co-authorship with one of the authors PB.
