# Supplementary material for: Pupil Size as a Gateway Into Conscious Interpretation of Brightness
Source: Front Neurol. 2018 Dec 13;9:1070. doi: 10.3389/fneur.2018.01070 (PMC6315169; doi:10.3389/fneur.2018.01070)
Supplement: Supplementary file 1 [file Data_Sheet_1.docx]

**Supplementary material**

## Figure S1

*Figure S1. Pupillary response to sun pictures and their phase-scrambled versions: same format as Figure 2 in the main text, except that trials are divided based on whether the sun/scrambled image was reported as seen (left) or unseen (right). A two-way ANOVA for repeated measures analyzed the effects of stimulus (sun or scrambled) and condition (seen or unseen), revealing a significant stimulus x condition interaction (F(1,19) = 11.37 p = 0.0032), and thereby showing that the sun vs. scrambled pupil difference varies significantly depending on stimulus awareness. Note that the different participants contributed to an unequal number of trials to the two conditions (given the inter-subject variability in CFS success rate); for this reason, it is important to confirm the result with the Linear-mixed-model approach (which accounts for this variability, and still shows a significant stimulus x condition interaction: F(1,1972) = 15.338 p<0.001).*

## Figure S2

*Figure S2. Luminance profile of the sun and phase-scrambled images as function of eccentricity (i.e. distance from the center of the image, where fixation was maintained). A. When averaging all 13 images, there is a tendency for sun images to be brighter than their phase-scrambled version near the fovea (note that sun images were anyway darker than the pre-stimulus screen, the luminance of which is indicated by the dashed blue line). We therefore selected 7 pairs of sun and scrambled images with matching luminance profiles (B) and selectively analyzed pupillary responses to this subset of images (Fig. S3).*

## Figure S3

*Figure S3. Pupillary response to the subset of sun and phase-scrambled pictures with matching luminance profiles (Fig. S2B); same format as Fig. S1. Also for this subset of trials, the two-way ANOVA for repeated measures analysing the effects of stimulus (sun or scrambled) and condition (seen or unseen) revealed a significant stimulus x condition interaction (F(1,17) = 9.90 p = 0.0059), confirming that the sun vs. scrambled pupil difference varies significantly depending on stimulus awareness even for this subset of trials. The Linear-mixed-model approach confirms this result, revealing a significant stimulus x condition interaction: F(1,1055) = 7.853 p<0.01).*
